# Reminiscences of the Insane

**Published:** 1887-05-07

**Authors:** 


### Reminiscences of the Insane.

By a Mental Physician.

(Continued from p. 13, vol. ii.)

If these Reminiscences require an apology, it is to be found in the practical conclusions which may be drawn from an experience of exceptional people. We shall not be misunderstood when we say that the first of these practical conclusions is, that the public mind must be taught to regard the subject of care and treatment, and moreover of precaution, as also exceptional. And clearly it further follows that the knowledge of those who undertake to advise or to instruct in matters of lunacy must be exceptional. Familiarity with the minute shades of difference between a man who is dangerous to the community or to himself from brain disorder, and the man who, although far from mentally sound, may be safely allowed to associate with the world, can alone determine what course should be pursued. Far be it from us to allege that even such familiarity will insure anything like an infallible judgment; all that we maintain is, that an exceptional familiarity with exceptional people must be better from this point of view than that want of familiarity which for the most part pertains to the general public, and it must be added, to the members of the legal profession. Much might be said, and said usefully, on this latter point, seeing that the mistake is so frequently made by learned lawyers, and especially by the judges of the land, of supposing that their knowledge of law entitles them to speak dogmatically as to the mental condition of prisoners who come before them. The absurdity of such a course is obvious, on considering how the parallel conduct of a physician, were he to interpret the English law to an English judge, would be regarded, and justly regarded, by those who sit on the Bench and give lessons from the Seat of Judgment to mental physicians who are at their mercy in the witness-box. But although a chapter might be written on "Judge and Doctor," we have no intention of pursuing the subject further in this place. Rather would we avail ourselves of the opportunity of expressing, in a journal devoted to the interests of hospitals, the importance of educating people to recognise the duty of attending to the early symptoms of mental disease, in order that they may receive prompt attention and skilled treatment.

*An Exceptional Disease requires Exceptional Knowledge.*

*Not Possessed by Lawyers.*

*Early Symptoms to be attended to.*

Much harm may be done by the irritation caused by well-meaning friends who mistake insanity and crime, mental disturbance and temper, delusion and unpardonable fancies, insane conduct and voluntary misbehaviour. Such a misunderstanding of a case, and the consequent friction between the insane and the sane members of a family, would be avoided by more extended knowledge of what constitutes that exceptional condition of the mind termed insanity, would lead to consultation with medical men, and the prevention of further conflict between relations or friends. It by no means follows—and here is the great mistake so frequently made—that a patient in an early stage of his disorder should be sent to a hospital. The course to be pursued depends upon a variety of considerations. Every case requires separate thought, and should be treated according to its own special merits. For one the asylum or hospital is the very best place, and would be much more likely to conduce to recovery than outside treatment. To another, however, a hospital would be absolutely injurious. It may be the best to place the patient in lodgings or in the house of a medical man, while in a few rare instances travelling abroad with a companion may offer the best chance of recovery.

*Different Cases demand different Kind of Care.*

A book might be written pointing out the popular errors of the public in regard to the insane. It is a proof of great advance to have completely changed the current of feeling in the course of a century in regard to those labouring under mental disorder. But although a most fortunate change of sentiment has rescued madmen from barbarous treatment, the very reaction has brought with it manifestations of ignorance which are unavoidable, constituted as man is, but not the less deplorable. These manifestations of emotional movements are to be deprecated, in the interests not only of the community, but of the unhappy patients themselves, on whose behalf an unfounded clamour is made. Nothing said in this place is intended to reflect for a moment upon the indignant exposure of abuses when these occur. Public opinion is often wrong, impulsive, and precipitant in its judgment, and to this extent is unjust; but, for all that, frequently as it blunders, it not unfrequently blunders on the right side, and it certainly cannot be dispensed with as regards the care of the insane and the oversight of those in whose power they are placed. The only difficulty is to keep it within due bounds when once called into activity. Instructed public opinion is of the greatest possible service in furthering the right management of hospitals, whether for the insane or the sane. In proportion, therefore, as popular errors are dissipated, the public mind will be profitably directed to the subject under consideration.

*Misguided Public Opinion.*

One of the popular errors in relation to the insane, and which underlies so many others, is the notion that a certain amount of criminality is attached to the mere circumstance of a person becoming deranged. This has partly arisen from the fact that some people do undoubtedly bring on an attack of insanity by their own misconduct; but the same occurs in purely physical disorders, without involving the like opprobrium. Criminality should not therefore be associated with insanity, either more or less than in bodily disorders; and no more stigma ought to attach to an attack of insanity than to an attack of pleurisy, indigestion, or gout. In fact, it is quite possible that less blame is deserved by one who labours under acute mania, than by the patient who has induced an attack of pleurisy by foolhardy exposure to cold, or who labours under indigestion from a gluttonous surfeit, or suffers the pangs of gout from the excessive imbibition of port wine.

*Insanity not Criminality.*

A grave popular error consists in the notion that vicious acts are almost necessarily the results of vice, unless the man is more or less of an idiot, or is stark mad in his conversation. Hence it is that in families in which an insane element arises, and in which the temper or moral conduct becomes changed, its true nature not being recognised, the misery of the household, so far from being mitigated, is on the contrary intensified by this change of character being referred to wilful sin. The intense suffering endured by such a family is only too well known by the physician to whom it is communicated—who, however, is frequently unable to remedy the mischief, although understanding its nature perfectly well, because the ignorance of others is a bar to the acceptation of his views and the line of action which he proposes. If on this point alone more general knowledge becomes diffused, the unhappiness of many a family will be lessened, and in some instances terminated.

Patient thought Vicious unless very Mad.

A cardinal error is delay in the treatment of cases of insanity, instead of promptly obtaining skilful medical advice. This promptitude may be called the A B C of the elementary lessons which the public require to learn. It constantly happens, however, that the countless and contradictory suggestions of friends and neighbours are listened to, and by turn adopted, until the disorder is more deeply rooted in the constitution; and when the advice of an experienced physician is sought, it is with much more difficulty eradicated, or is found, it may be, altogether beyond the skill of medicine and moral management to remove. Again, evil is wrought not unfrequently by the friends of patients interfering with the treatment, and by their removal from care before the right time has arrived. This is often done under protest from the physician in charge, with disastrous results to either the patient or his family—very likely to both. Quite recently, the wife of a patient insisted upon complying with his request to remove him from the Asylum, in the teeth of the strong advice of the Superintendent. The result was that within a very short period he murdered his wife.

Delay in Treatment.

Premature Removal from Asylums.

Popular errors in regard to insanity prevent the possibility of prompt action being taken in placing a number of dangerous persons under care who are on the brink of committing acts of violence in consequence of mental instability and insane impulses, but who have not committed as yet any overt act, and consequently are not subjected to any restriction of their freedom. There is the jealousy of interference with the liberty of the subject prevalent amongst us. Here it is absolutely impossible to take action until public opinion is levelled up to a much higher point of knowledge and the recognition of cerebre-moral disease than it now is. Only the other day certificates of lunacy had been actually obtained for the purpose of placing a gentleman in an asylum, when the unfortunate prejudice against confinement induced the family to delay action. The consequence was that within a few days, the patient mutilated his wife in a frightful manner, destroying her life, and then committed suicide himself.

Dangerous Persons at Large.

Fearful Consequences.

Were we to include under popular errors the ignorance which not only does mischief when insanity has developed itself, but is so fruitful a cause of its production, we should have to enter upon the wide subject of the prophylaxis of insanity. This is not our intention. Suffice it to say in this place that the prevention of mental disorder ought to extend in proportion to the knowledge of its causes, but that in addition to this, experience only too truly proves that necessary as is knowledge, something more is needful, but too often absent—the courageous determination to carry this knowledge into practice.

(*To be continued.*)

